# Clinical applications of exercise stress echocardiography in the treadmill with upright evaluation during and after exercise

**DOI:** 10.1186/1476-7120-11-26

**Published:** 2013-07-22

**Authors:** Carlos Cotrim, Isabel João, Paula Fazendas, Ana R Almeida, Luís Lopes, Bruno Stuart, Inês Cruz, Daniel Caldeira, Maria José Loureiro, Gonçalo Morgado, Hélder Pereira

**Affiliations:** 1 Cardiology Department, Garcia de Orta Hospital, Avenida Torrado da Silva, 2805-267 Almada, Portugal

**Keywords:** Exercise stress echocardiography, Treadmill, Orthostatic evaluation, Pulmonary hypertension, Mitral stenosis, Intra-ventricular gradients, Athletes, Cardiac X syndrome, Hypertrophic cardiomyopathy, Aortic stenosis

## Abstract

Exercise stress echocardiography is the most frequently used stress test in our laboratory. Exercise echocardiography is used mainly in the study of patients with coronary artery disease. However, the technique is increasingly being used to study other diseases.

In our centre, we use an original methodology, published by us in 2000, in which we evaluate heart function during exercise in the treadmill. After the exercise, patients are maintained in orthostatic position when appropriate or lying down in left lateral decubitus for further evaluation. Since this method seems to increase the quality and the quantity of information obtained in so many clinical arenas, we now present a detailed review of this methodology and its applications.

## Introduction

The high prevalence of coronary artery disease has led to the development of reliable and accessible non-invasive diagnostic techniques. Among these, stress echocardiography has been accepted as a valuable method in the detection of myocardial ischemia [1-8] outweighing the limitations imposed by the widely used treadmill stress test. In our center, stress echocardiography is preferentially performed using a treadmill exercise protocol. Pharmacological stress echocardiography is only used to evaluate myocardial viability, or when patients cannot exercise adequately.

### Methodology of exercise stress echocardiography

#### Exercise test in treadmill

Firstly, patients are questioned about their symptoms, past cardiovascular medical history and risk factors for coronary artery disease. After an explanation and preparation for the procedure by a cardiopulmonary technician, a 12-lead electrocardiogram is obtained. Bruce protocol is usually performed. In the assessment of non-coronary artery disease, a modified Bruce protocol is applied for easier evaluation of Doppler parameters rather than the classical Bruce protocol, in some clinical scenarios.

Criteria for test interruption are: fatigue, angina with increasing intensity, dizziness, ST-segment depression greater than 3 mm, complex ventricular arrhythmias, systolic blood pressure greater than 240 mmHg or diastolic blood pressure greater than 130 mmHg or a blood pressure drop greater than 20 mmHg. The test is considered to be positive for myocardial ischemia when ST-segment depression occurs, with a horizontal or down sloping displacement greater than 1 mm measured 0.08 seconds after the J point. The ECG exercise treadmill test is considered inconclusive when there are baseline ST-T changes (left bundle branch block, digitalis effect, left ventricular hypertrophy) or when the patient does not reach 85% of the theoretical maximum age-adjusted heart rate. The exam is negative for myocardial ischemia when the patient’s heart rate exceeds 85% of the theoretical maximum age-adjusted heart rate without the previously mentioned changes.

### Exercise stress echocardiography

Exercise stress echocardiography is performed in our center since 1996. This method allows the evaluation of cardiac function, rather than electrical activity, during exercise. Peteiro et al. have firstly published imaging acquisition during exercise in 1999 [6] and one year after we published a detailful methodology article[7] and most recently other centers have also described it [8,9].

**Figure 1:**
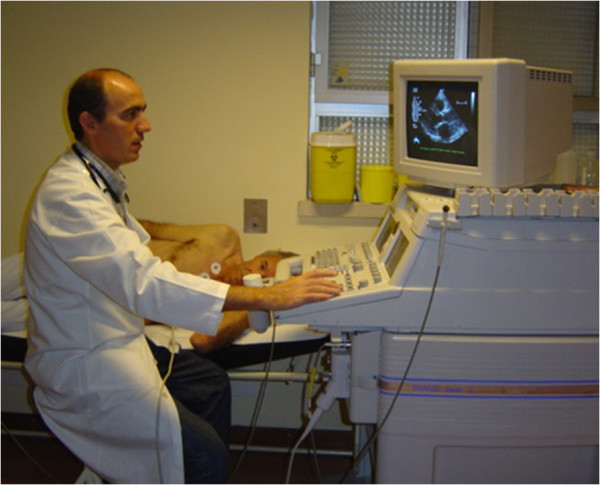
Echocardiographic data acquisition with the patient in left lateral decubitus before exercise.

Basically, before starting the exercise test, a baseline echocardiogram is performed in the left lateral decubitus position (Figure 1) for initial assessment, with 2D and M-mode image acquisition in at least four planes: parasternal long axis, parasternal short axis, apical four-chamber and apical two-chamber; with Doppler parameters being evaluated and stored according to the patient’s disease. These were re-evaluated if possible during the first minute before starting the exercise stress treadmill test in the standing position.

**Figure 2:**
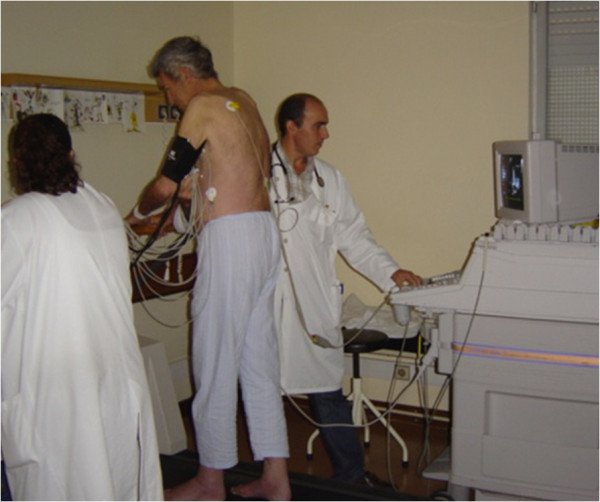
Echocardiographic data acquisition with the patient in orthostatic position during exercise in treadmill.

In our center, 2D echocardiography is also performed in the standing position throughout the exercise test (Figure 2, see Additional file 1 and 2) with image acquisition at exercise peak.

After stopping the exercise test the patient is quickly placed in the left lateral decubitus position and images are again acquired in the previously referred planes (Additional file 3). In some circumstances, for example for the detection and evaluation of intra-ventricular gradients in hypertrophic cardiomyopathy, the patient is kept upright after finishing the stress test and echocardiogram carried out at this position. As for the evaluation of left ventricular regional wall motion abnormalities, we use the American Society of Echocardiography model that divides the left ventricle into 16 segments [10]. Ischemic changes are considered when segments develop hypokinesia, akinesia or dyskinesia. Nevertheless, when an akinetic segment becomes dyskinetic it is not considered to be ischemic.

The studies with the method were approved by the Ethics Committe at Garcia de Orta Hospital.

### Applications of exercise stress echocardiography

#### Ischemia detection

Stress echocardiography has demonstrated great utility in ischemia detection because of its high sensitivity and specificity [7,11-14] both in patients without a history of prior intervention and in those previously submitted to percutaneous coronary intervention [15] or coronary artery bypass graft surgery [16]. Females have a higher rate of false-positive results with exercise electrocardiographic testing. In this population, stress echocardiography demonstrated to be an accurate method for ischemia detection [17]. In patients with left ventricular hypertrophy, stress echocardiography has a sensitivity of 84% and a specificity of 75% in the detection of ischemia [18], justifying its use in clinical practice. Detection of ischemia with exercise electrocardiographic testing is limited in patients with left bundle bunch block. Exercise stress echocardiography may also face some limitations in these patients because of the paradoxical motion of the inter-ventricular septum. In a series of 30 patients with left bundle branch block (LBBB), Pellika and colleagues showed that exercise stress echocardiography had 60% sensitivity in ischemia detection, compared to 88% sensitivity with dobutamine stress echocardiography [19]. Other authors evaluated 35 patients with LBBB, using treadmill exercise stress echocardiography found higher sensitivity (76%), with specificity and accuracy of 83% and 80%, respectively [20]. This topic was also addressed in a meta-analysis which revealed that exercise ECG and myocardial perfusion imaging had the highest sensitivity, while stress echocardiography had the highest specificity, on ischemia detection in patients with LBBB [21]. The prognostic accuracy of myocardial perfusion and stress echocardiography appeared similar. Although our conclusions are based in a small sample study, dobutamine stress echocardiography is preferred to exercise stress echocardiography in this subgroup of patients in our center. Detection of ischemia, as well as its magnitude, has obvious prognostic implications. In a study with more than 500 patients [22], Marwick demonstrated that the use of stress echocardiography to detect ischemia provides additional prognostic information. The comparative advantage of exercise echocardiography with image acquisition during treadmill exercise was clearly demonstrated by Peteiro [23,24], who showed an increased diagnostic accuracy with this methodology compared with evaluation only before and after exercise. His results confirm our preliminary results from a previous small study [25]. In comparison with other widely available imaging techniques, exercise stress echocardiography has many advantages. These include greater safety, with only one adverse event in every 7000 exams, when compared to one adverse event in every 700 dobutamine stress echocardiograms [26]. This was addressed by the international practice guidelines [27], that reserve drug-induced stress echocardiography for those unable to perform an exercise stress test. Another advantage of exercise stress echocardiography is the radiation-free nature.

### Evaluation of patients with suspected or confirmed pulmonary hypertension including patients with mitral stenosis

**Figure 3:**
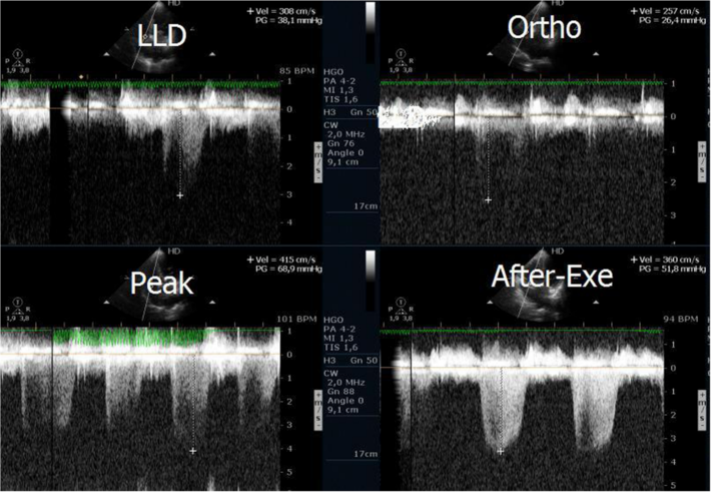
Right ventricular/ right atrium gradient, evaluated with CW Doppler, at different stages of the study in one patient with mitral stenosis.

**Figure 4:**
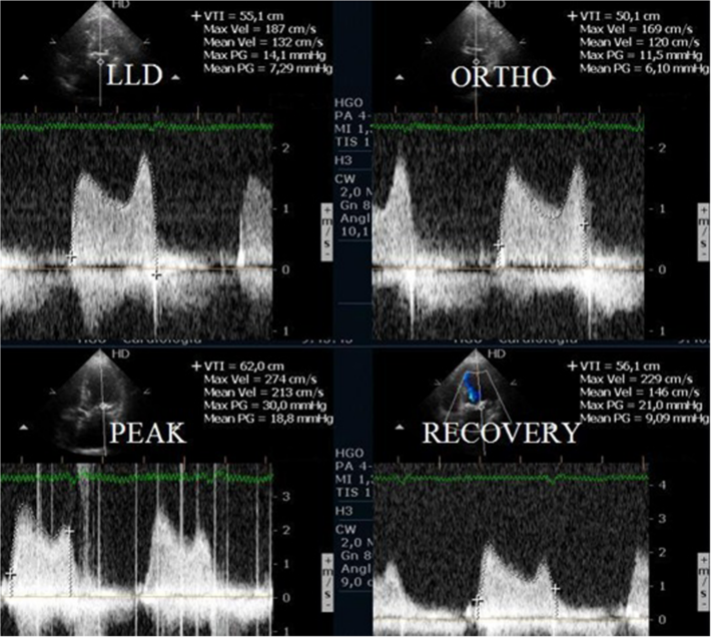
Left atrium/ left ventricle gradient, evaluated with CW Doppler, at different stages of the study in one patient with mitral stenosis.

Evaluating pulmonary artery systolic pressure at rest using echocardiography is common practice and of considerable clinical importance [28]. A diagnosis of pulmonary arterial hypertension was based on a mean pulmonary artery pressure of >25 mmHg at rest or >30 mmHg during exercise [29]. Although routine assessment is generally carried out only at rest, the clinical importance of determining pulmonary artery systolic pressure during exercise has been demonstrated in various clinical conditions, particularly in mitral stenosis [30-33], mitral regurgitation [34] and rheumatologic disease [35]. Our group has published a study with patients having mitral stenosis. We assessed the right ventricle-right atrium (RV-RA) gradient using continuous wave Doppler in four stages: 1) left lateral decubitus before exercise testing; 2) in standing position; 3) at peak workload before termination of the test; 4) in the first 60 seconds of the recovery period in left lateral decubitus (Figure 3). The mean gradient between the left atrium and left ventricle was also determined at the different stages of the test in patients with mitral stenosis (Figure 4) (Additional file 4). We compared the variation of the gradient values between right ventricle and right atrium obtained at peak workload with those obtained only in the immediate recovery period. In this group of 56 patients with mitral stenosis, the decision to treat based on pulmonary artery systolic pressure of >60 mmHg determined by peak work load gradients between right ventricle and right atrium [30,36] resulted in a positive net reclassification of 10 patients (18% of those with this pathology). These patients would have continued with medical therapy if the decision had been based on the values obtained during the recovery period, although peak overload RV-RA gradient measurement led to valvuloplasty or valve replacement referral. The results obtained for left atrium/left ventricle gradient influenced clinical decisions in three patients only, in whom no RV-RA gradient was detected at peak workload.

The same methodology was used to decide whether to refer 42 patients with systemic sclerosis for right heart catheterization. Results pointed that a significantly increase of peak workload RV-RA gradient resulted in 13 more patients (30% of those with this pathology) being referred for this procedure compared to a strategy that considers gradient values obtained only during the recovery period.

**Figure 5:**
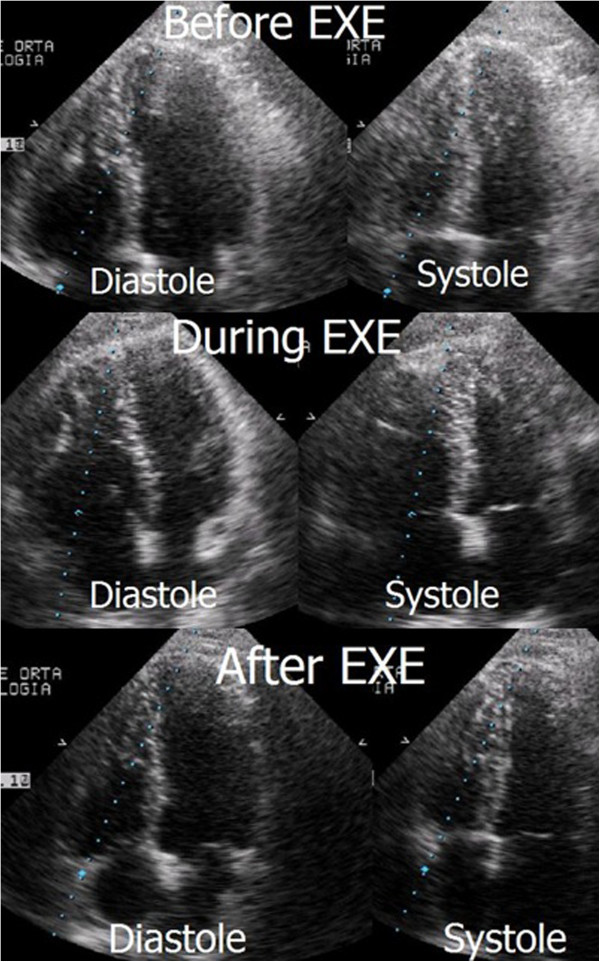
Apical four chamber view before, during, and after exercise with right ventricle dilatation only visible during exercise.

In patients with previous history of pulmonary thromboembolism, with mild or moderate pulmonary hypertension or with unclear cause for symptoms, performing exercise stress echocardiography may help in clinical evaluation and decision. We have published two case reports that highlight such importance, as exercise test may unmask right ventricular dilation, with baseline and post-exercise echocardiography without chamber dilation (Figure 5 and Additional files 5, 6, 7) [37,38]. After further evaluations these two patients have been submitted to pulmonary thromboendarterectomy. These cases underline the clinical value of echocardiographic evaluation also during exercise.

In patients with severe pulmonary hypertension (PH), exercise echocardiography may improve our knowledge of PH pathophysiology [39]. In this setting RV-RA gradients did not decrease in the standing position and rose significantly with orthostatic isotonic exercise during exercise. Pulmonary artery systolic pressure reaches suprasystemic values, stroke volume and systolic blood pressure did not rise during exercise in patients with severe pulmonary hypertension. Patients with a decrease in stroke volume had worse clinical evolution [39].

**Figure 6:**
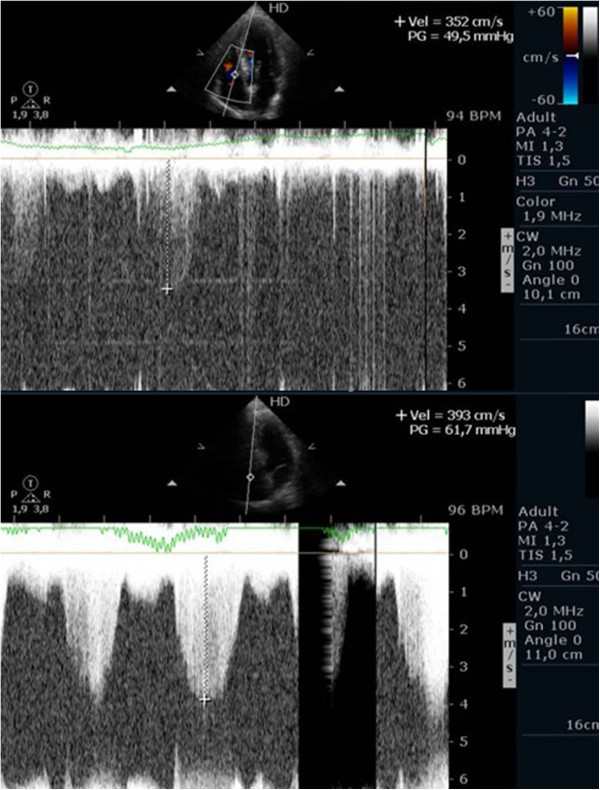
Continuous wave Doppler signal of the tricuspid regurgitant jet at peak exercise before and after injection of contrast.

RV-RA gradients may be underestimated in patients whose acoustic windows pose difficulties to obtain adequate tricuspid regurgitation Doppler signals. The use of air-blood-saline contrast during exercise has led to improvement of Doppler signal in several clinical contexts (Figure 6) [40]. Therefore, the use of contrast should probably be limited to the patients with very poor tricuspid regurgitation jet signal, to obviate the apparently high number of false positives results that we found. The contrast increased Doppler signal, in patients with previous optimal regurgitant jet signal, can significantly increase RV-RA gradient to values greater than 40 mmHg, without a clear clinical meaning. Thus, contrast should be used as an aid to obtain a measurable RV-RA gradient, but should not be used routinely in all patients submitted to this exam.

### Intra-ventricular obstruction induced by exercise in athletes with “positive screening” in medical evaluation for sports practice

The development of intra-ventricular gradients (IVG) during exercise is rare, and it usually occurs in association with left ventricular hypertrophy [41,42]. The development of significant IVG during exercise has been anecdotically described in athletes [43-45], but the clinical impact of this observation and the most appropriate exercise technique (upright vs. semi supine) to provoke IVG remains unknown. Supine position is less technically demanding but also less physiological than upright exercise. Treadmill exercise stress echocardiography is usually, performed in the post-exercise phase with the patient in the supine position. In our center, the echocardiography is performed in the orthostatic position during all the stages of the exercise test [7].

It should also be noted that in normal daily life, after exercise, athletes do not assume supine position, in opposition to the post-exercise evaluation done in most of other centers. Such serial measures in upstanding position (at peak overload and after exercise) were emphasized in a study enrolling 139 young athletes (mean age 22 years; 135 amateur and 4 professional) with positive screening according to the European Society of Cardiology guidelines [46], with a normal echocardiogram at rest without left ventricular hypertrophy or significant valve disease [47]. One hundred and twelve (81%) had symptoms (chest pain, dizziness or syncope) or positive exercise ECG (electrocardiogram) treadmill testing (11 athletes). Regarding the 27 asymptomatic athletes: four had family history of sudden death, three had mild mitral valve prolapse without mitral regurgitation, 18 had ECG repolarization abnormalities and three had ventricular premature beats on the ECG.

About 37,4% of the athletes (52 athletes) developed IVG (greater than 50 mmHg) and 62.6% (87) did not develop IVG (greater than 50 mmHg).

**Figure 7:**
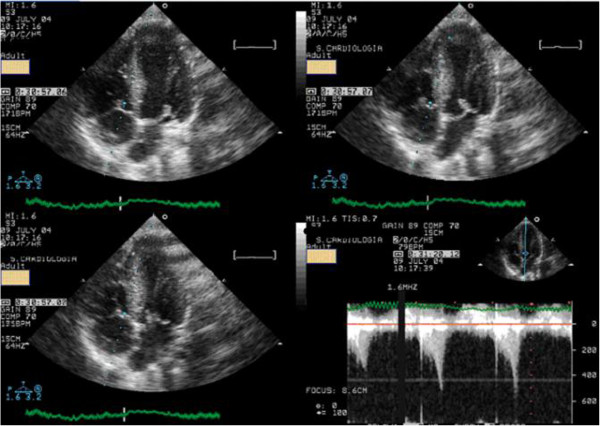
Systolic anterior movement of mitral valve and significant intra-ventricular gradient detected at peak exercise.

Among those who developed IVG, 63% (23 athletes) developed systolic anterior movement of mitral valve (SAM) (Figure 7, Additional file 8) associated to a significant IVG during exercise (Figure 7, Additional file 9). IVG was present in all athletes at recovery in orthostatic position. Remarkably, in 7 of these IVG was only present at this stage.

It has long been known that small magnitude intra-ventricular gradients are a common phenomenon. Three mechanisms have been proposed [48] to explain the circumstances when they significantly increase during exercise: 1) increase in non-obstructive physiological gradients; 2) end-systolic obstruction secondary to ventricular mid-cavity obliteration; and 3) mid-systolic obstruction caused by SAM of the mitral valve restricting ejection. However, SAM usually occurs when there is some change of the ventricular chamber geometry or in the mitral valve apparatus. This was not the case of our athletes, although it has been demonstrated that intra-ventricular gradients can be caused by manoeuvres that change loading conditions in structurally normal hearts [49], such as those that occur during competitive sport practice.

Sudden death in young athletes has been thoroughly studied, and there is an agreement that the most frequent causes are hereditary or congenital [50]. However, in some of the series [51], around 30% of autopsy studies show no abnormality, which suggests that the standard screening programs are failing to prevent sudden death.

The morphological study of the hearts of the patients included in our study revealed no abnormalities [47]. The phenomenon that we detected before, during and after exercise testing, in orthostatic position – intra-ventricular gradient associated with mitral valve SAM at peak and after exercise – could well have been responsible for the positive screening in the athletes from this group.

The medical examination of these athletes was carried out mostly because of symptoms arising from strenuous exercise. Symptoms were not reproduced during exercise in most of the athletes, however possible symptom-related cardiac function abnormalities were observed [42-45]. These were more common during and after exercise in orthostatic position (41 and 52 athletes, respectively), however there were a few athletes whose abnormalities were present before exercise (2 athletes). This phenomenon is not amongst the diagnoses that contraindicate participation in competitive sport, according to the recommendations of the 36^th^ Bethesda conference [52] and the European Society of Cardiology [46]. However, it is possible that the phenomenon described in these athletes could be amongst the causes of sudden death in cases where anatomopathological examination reveals no abnormalities, and we accordingly referred them for assessment to a sports medicine centre. In our opinion, the cases described, in which significant abnormalities in cardiac function were found before, during, and more clearly after exercise in orthostatic position, suggest that this methodology, may be useful, if applied to the athletes that have symptoms related to exercise but no structural abnormalities. We should note that this phenomenon has been almost excluded to be a normal response to exercise in healthy adults [53,54]. The results of our study [47] contribute to the importance that the literature begins to attribute to the search of intra-ventricular gradients, as a possible cause of symptoms related to effort in athletes and also in paediatric population [55,56].

The possible association between the development of IVG during exercise and symptoms was described before [42-46,57,58] and our results seams to reinforce this association.

#### The orthostatic factor

The occurrence of intra-ventricular gradients during exercise in symptomatic athletes is a frequent finding. The cases that we presented more recently have the particularity that the recovery has been done in orthostatic position – as it happens in the daily activities of athletes – and the gradients were similar to those in patients with hypertrophic cardiomyopathy that we studied before [59].

**Figure 8:**
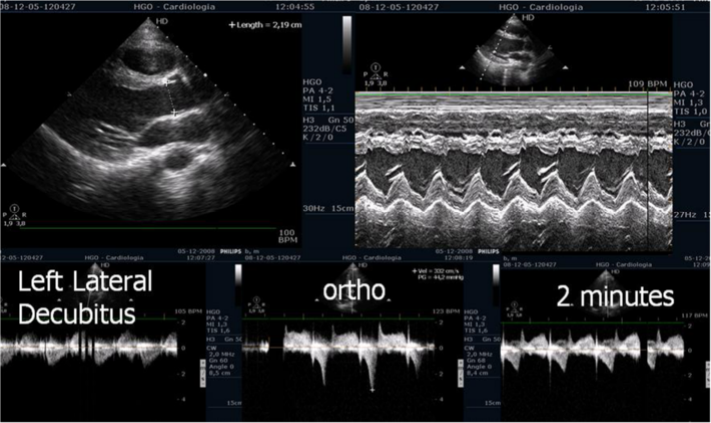
Intra-ventricular gradient present in orthostatic position before exercise in one athlete decreases in the initial phase of exercise test.

**Figure 9:**
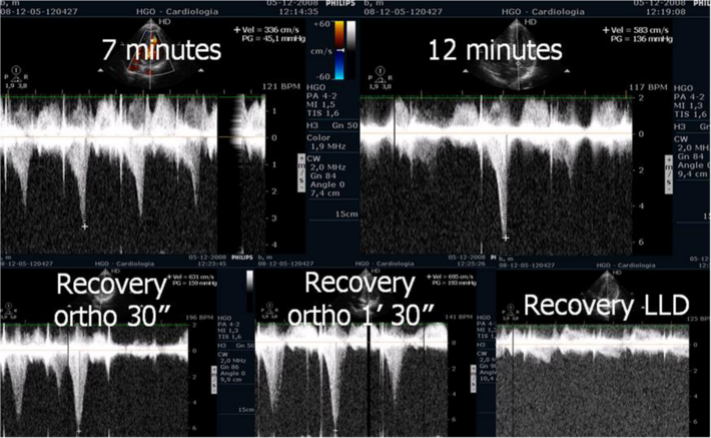
**Intra-ventricular gradient increases during the last part of exercise test and after exercise in orthostatic position.** Obstruction suddenly disappears putting the athlete in decubitus.

For the very first time, in two athletes, we also described the possibility that individuals without left ventricular hypertrophy develop intra-ventricular gradients when in orthostatic position before exercise, as was described before in hypertrophic cardiomyopathy patients [59]. In the beginning of exercise (Figure 8) the gradient initially decreased, possibly because the contracting lower limbs muscles increased preload significantly. The orthostatism after exercise causes a greater decrease in preload than compared to the supine position (Figure 9) [7]. This is most probable cause of the IVG increase in most of the athletes and may explain the orthostatic recovery IVG documented in 7 athletes. Nevertheless we cannot forget that other factors such dehydration during sports practice, increase the risk of significant IVG.

Thus, continuous wave Doppler constitutes a new step in the use of stress echocardiography as a diagnostic tool, beyond the common indications such as coronary heart disease [60], and includes the use of a new stressor: orthostatism [47,59].

Therefore, we suggest that exercise stress echocardiography, with evaluation in upright position before, during and after exercise, should be part of a new diagnostic algorithm whenever athletes have positive screening on medical evaluation, particularly those with symptoms.

### Monitoring the use of beta blockers

**Figure 10:**
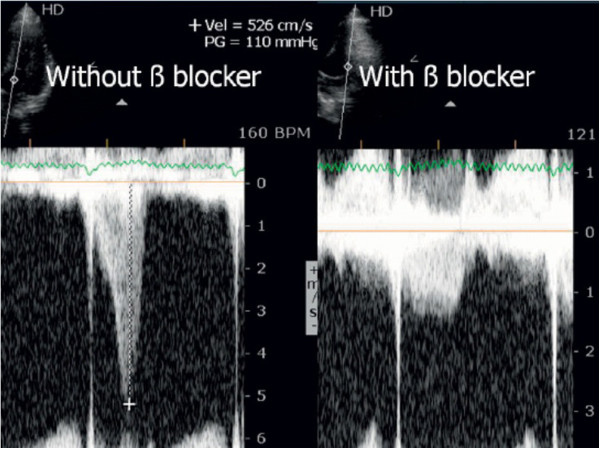
IVG in one athlete assessed before and during beta blocker therapy.

Most of the athletes evaluated in the previous study have been treated with beta blockers by their assistant physicians. We conducted an open-label, prospective, non-randomized study to provide proof of concept that exercise stress echocardiogram can be a guide to tailored treatment in athletes with positive screening [61] and that develop IVG and mitral valve SAM on exertion. We evaluated 52 athletes that developed IVG and 35 (32 had exercise-related symptoms or positive exercise electrocardiography) repeated the exercise stress echocardiogram under treatment with ß blockers Thirty athletes (85%) showed improvement with a significant reduction of IVG and of the prevalence of SAM were shown (Figure 10). These changes were associated to a significant reduction in heart rate at peak exercise. We concluded that athletes with positive screening - mostly by symptoms - for sports practice and IVG on exertion, treatment with oral ß blockers prevented the occurrence of IVG and SAM or significantly reduced its magnitude.

Exercise echocardiography provides a useful tool to identify athletes with positive screening and normal rest echocardiogram who may benefit from beta-blocker therapy. This has also been observed by other authors [62].

### Intra-ventricular gradients in patients with cardiac X syndrome

The development of IVG during dobutamine stress echocardiography has been largely reported and this finding is commonly associated with symptoms during stress [63,64]. The occurrence of IVG during exercise stress echocardiography is rarely found [41]. In a group of 10 patients who developed IVG during dobutamine stress echocardiography, we performed exercise stress echocardiography and we found a small magnitude IVG in only one of them [65]. But in a 23 years old male, with a positive treadmill test, a structurally normal heart and normal coronary angiography, an exercise stress echocardiography was performed and during the study we unexpectedly detected a 102 mmHg intra-ventricular gradient (Additional file 9) [43] and systolic anterior movement of the mitral valve (Additional file 8). A similar case has been reported previously [42] and treated successfully with beta-blockers. After our first case, published in 2002 [43], we have conducted one large study to assess intra-ventricular gradients during exercise stress echocardiography in patients with angina, positive stress electrocardiography, normal coronary arteries, and normal echocardiogram (cardiac X syndrome) [66]. We enrolled 91 patients (48.3% women), with angina, positive exercise ECG treadmill testing (four patients had ischemia detected in a myocardial perfusion study), normal rest echocardiogram with left ventricular hypertrophy, and absence of coronary artery disease after coronary angiography procedure.

Thirty-three patients (36%) developed IVG (Figure 7e Additional 9). In these patients, IVG at peak exercise was 86 ± 34 mmHg (30 to 165 mmHg) and 23 pts (70%) developed SAM (Figure 7, Additional file 8) during exercise, associated with IVG. No patient developed segmental wall abnormalities. The results of our study, in which 36% of the patients with normal coronary angiogram and positive treadmill exercise test developed significant intra-ventricular gradients, suggest that ST-segment depression may be related with the development of IVG during exercise, which is possibly involved in the genesis of electrocardiographic changes. The possible association between cardiac X syndrome and IVG during exercise was described before [57,58], however the inclusion of patients more prone to develop IVG such as hypertensive ones with left ventricular hypertrophy limits their findings [41].

The full-length of exercise imaging as mentioned before may have contributed to the high number of patients that developed SAM of the mitral valve in association with IVG, compared to other authors’ data [57,58] (Additional file 1 and 2) [7]. It could have also contributed to the increased magnitude of the IVG.

We concluded that a significant proportion of patients with cardiac X syndrome develop significant intra-ventricular gradients during exercise. The authors believe that this phenomenon may constitute a new entity, that might explain a proportion of the heterogeneous group of patients with angina, ST-depression during treadmill exercise test and normal coronary angiography. Exercise stress echocardiography may provide additional information whenever patients fulfil criteria of cardiac X syndrome.

### Evaluation of patients with hypertrophic cardiomyopathy

**Figure 11:**
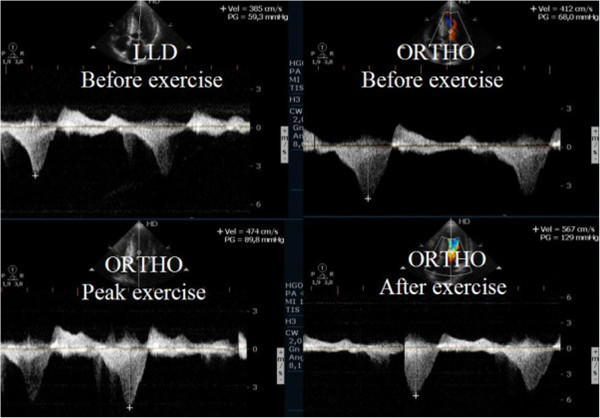
Intra-ventricular gradient present in all the phases of the study in one patient increasing also after exercise in orthostatic position.

**Figure 12:**
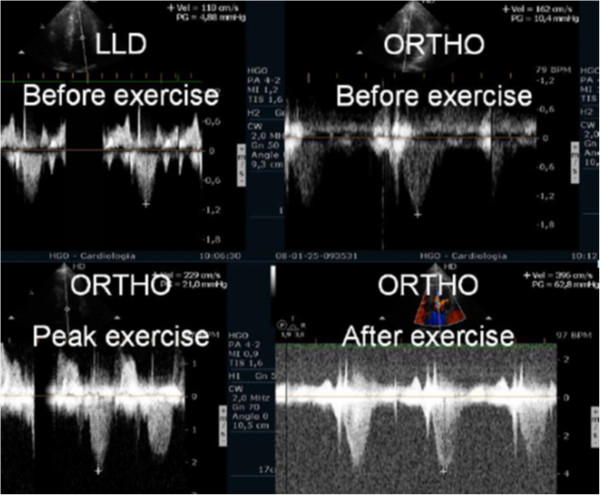
Intra-ventricular gradient present only in the recovery and in orthostatic position.

Left ventricular outflow tract obstruction is the major cause of symptoms in hypertrophic cardiomyopathy (HCM) and it has been associated to worse prognosis [67]. It is present in one-third of the patients at rest while the remaining two-thirds can be provoked [68,69], nevertheless the best stress protocol is still yet to be defined. In our center, exercise echocardiography with image acquisition during treadmill exercise (considered to reflect exercise during daily activities) is commonly used in the evaluation of patients with HCM, enabling assessment of outflow gradient during physiologic exercise [7]. In these patients LVOT gradients increased in from supine to orthostatic position, and continued to augment at peak exercise, however after exercise the gradient decreased rapidly when measured in left lateral decubitus; the assessment of intra-ventricular gradient in recovery period in supine position probably does not reflect changes occurring immediately after effort [70] or the pathophysiology of this condition [68,69,71]. In a case report we stated that in one patient with HCM, the intra-ventricular gradient continued to increase if we maintained the patient in orthostatic position after exercise (Figure 11) (Additional files 10 and 11). Taking this into account, we performed a pilot study in 17 patients with HCM (11 with gradient above 30 mmHg under resting conditions) to determine the impact of orthostatism in the development of IVG in HCM [59]. Three patients without resting obstruction developed a significant intra-ventricular gradient during exercise; one patient only developed such a gradient in the recovery period in orthostatic position (Figure 12); two patients had neither resting nor exercise-induced obstruction. All patients with obstructive HCM increased IVG in orthostatic recovery. These results are different from those observed in our initial study [68] and from other studies in which the patients assume the supine position immediately after exercise [9,72-76]. We have most recently expanded this observation to a cohort of 51 non-obstructive HCM patients, where similar results were obtained. The importance of orthostatic position in this particular group of patients as an additional and new stressor has also been underlined by other investigators [77-83]. All that considered this method may enhance the ability to evaluate patients with HCM and understand the mechanisms of symptoms. Laying the patients in supine position after any type of exercise may be meaningless from the clinical point of view, because this does not happen in real life. Future guidelines should clearly recommend a single methodology to be employed.

### Aortic stenosis

**Figure 13:**
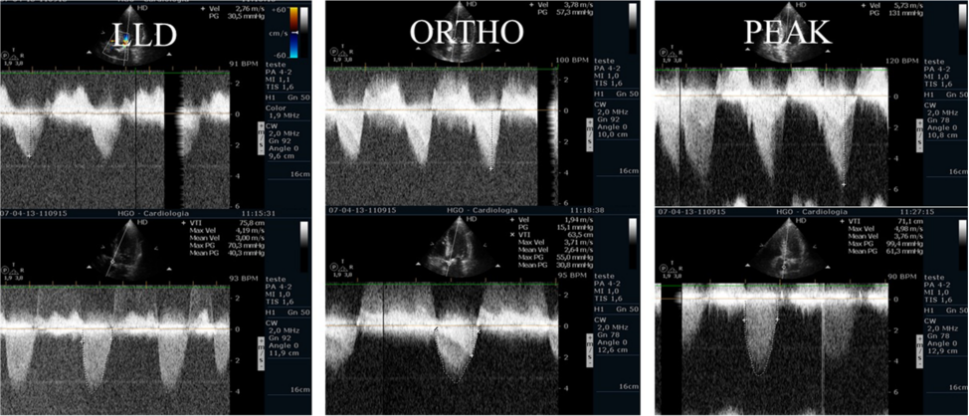
Intra-ventricular gradient present in all the phases of the study in one patient with symptomatic aortic stenosis increasing also after exercise in orthostatic position.

**Figure 14:**
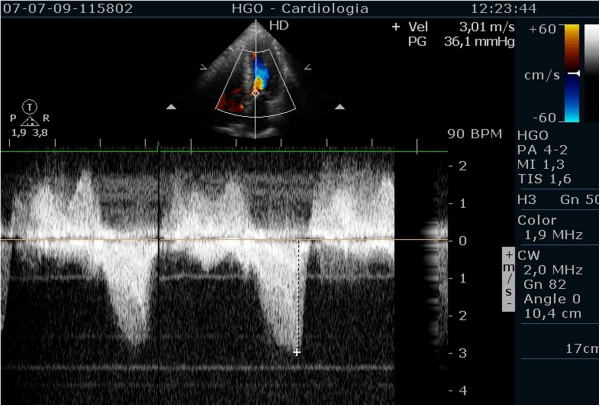
Intra-ventricular gradient evaluated in the same patient during beta blocker therapy.

A considerable proportion of the recommendations in the guidelines of valvular heart disease management have low levels of evidence, highlighting the need for further clinical investigation in this area [31]. In valvular heart disease, exercise testing is preferred over pharmacological stress because it provides insights regarding exercise related symptoms and blood pressure responses [36]. Supine bicycle exercise is recommended because Doppler information can be obtained during the different stages of exercise [27], rather than post-treadmill imaging, when there are substantial and rapid changes in heart rate and loading conditions. In our center we perform echocardiography during exercise in the treadmill (Additional files 1 and 2 and 4) as Peteiro group also does [7,8,84,85]. Supine exercise is not as physiologic as treadmill exercise, the equipment is not as widely available as the treadmill and the maximum VO2 attained is at least 10% lower with bicycle [86]. We use exercise stress echocardiography in the evaluation of patients with asymptomatic moderate and severe aortic stenosis. Following the literature [36,87-89], patients with an increase of 20 mmHg in the aortic mean gradient may be considered for surgery referral. The guidelines of European Society of Cardiology [31] on valve pathology consider that exercise echocardiography has the potential to give prognostic information but is not recommended because is not widely accessible, could be technically demanding, and requires specific expertise. About a third of the asymptomatic patients with severe aortic stenosis submitted to exercise test are symptomatic and to the best of our knowledge complications did not occur in this group of patients [87-90]. In our centre we perform exercise stress echocardiography in selected symptomatic patients to better clarify the mechanism of symptoms. As an example, we have published the case of a symptomatic patient in which the symptoms were probably due to the development of an significant intra-ventricular gradient [91], (Figure 13) due to SAM of mitral valve (Additional file 12) and was then treated with beta blocker and then become asymptomatic (Figure 14). This kind of patients with severe aortic stenosis in which proof is done that the symptoms have another cause should possibly be better labelled as patients with hemodinamicaly severe aortic stenosis with symptoms caused by other mechanisms.

### Limitations of the methodology

The development of a new stress echocardiography protocol should be accomplished by a multicentric validation study. In our center we established that autonomous practice of exercise stress echocardiography need at least 100 supervised exams. Our results were reassuring for the benefits and advantages of exercise stress echocardiography at least in the search for intra-ventricular gradients on HCM, in athletes with positive screening in medical evaluation for sports practice and in patients with X syndrome. Nevertheless, further studies are needed to evaluate in a powered sample size the value added of this new method in different conditions.

## Conclusions

The low cost, safety, diagnostic accuracy, possibility of evaluation of functional capacity and lack of radiation should make exercise stress echocardiography a first line procedure for patients with suspected or confirmed coronary artery disease. The possibility of evaluation of Doppler data during and after exercise, including in orthostatic position if appropriate, with the extraordinary amount of information that can be obtained, makes its use imperative in patients with hypertrophic cardiomyopathy, in athletes, in syndrome X patients, in patients with pulmonary hypertension and in patients with valve disease.

## Consent

Written informed consent was obtained from the patients for the publication of this report and any accompanying images.

## Abbreviations

IVG: Intra-ventricular gradients; ECG: Electrocardiogram; SAM: Systolic anterior movement; LVOT: Left ventricular outflow tract; HCM: Hypertrophic cardiomyopathy; VO2: Oxygen consumption; RV-RA: Right ventricle-right atrium; PH: Pulmonary hypertension.

## Competing interests

The authors declare that they have no competing interests.

## Authors’ contributions

CC reviewed literature and wrote the manuscript, performed exercise echocardiography with the method described in the article. IJ, PF, ARA, LL performed exercise echocardiography with the method described in the article, participated in drafting the article, and revised the manuscript for important intellectual content. BS, IC, DC, GM, MJL, HP participated in drafting the article, and revised the manuscript for important intellectual content. All authors read and approved the final manuscript.

## Supplementary Material

Additional file 1 Echocardiographic evaluation during exercise. Video, showing how the echocardiogram is done during exercise. Click here for file

Additional file 2 **Echocardiographic evaluation during exercise.** Video, showing how the echocardiogram is done with particular focus on hand positioning with cubital border attached to the patient thorax. Click here for file

Additional file 3 **Positioning the patient in left lateral decubitus after exercise.** Video, showing how the echocardiogram is delayed to put the patient in left lateral decubitus. Click here for file

Additional file 4 **Images obtained during exercise test in one patient with mitral stenosis.** Images obtained during the exam. Mitral flow is easily observed during exercise echo. Click here for file

Additional file 5 **Echocardiographic images obtained before exercise in one patient with thromboembolic pulmonary disease.** Apical four chamber view obtained in apical window before exercise where we can see normal dimension of right ventricle. Click here for file

Additional file 6 **Echocardiographic images obtained during exercise in one patient with thromboembolic pulmonary disease.** Apical four chamber view obtained in apical window during exercise where we can see dilatation of right ventricle. Click here for file

Additional file 7 **Echocardiographic images obtained after exercise in one patient with thromboembolic pulmonary disease.** Apical four chamber view obtained in apical window after exercise where we can see again normal dimension of right ventricle. Click here for file

Additional file 8 **Echocardiographic two dimensional images obtained during exercise.** Apical four chamber view obtained in apical window during the final phase of exercise containing two dimensional data with SAM of mitral valve. Click here for file

Additional file 9 **Echocardiographic continuous Doppler images obtained during exercise.** Intra-ventricular gradient evaluated continuously in one athlete with symptoms. Click here for file

Additional file 10 **Echocardiographic images obtained before exercise in one patient with non-obstructive hypertrophic cardiomyopathy.** Apical four chamber view obtained in apical window before exercise in one patient with non obstructive HCM containing two dimensional data (with SAM). Click here for file

Additional file 11 **Echocardiographic images obtained after exercise in the same patient in orthostatic position.** Apical four chamber view obtained in apical window after exercise in the same patient with SAM causing severe obstruction. Click here for file

Additional file 12 **Echocardiographic images obtained during exercise in one symptomatic patient with aortic stenosis.** Apical four chamber view obtained in apical window during the final phase of exercise containing two dimensional data (with SAM). Click here for file
